# “It saved me from the emergency department”: A qualitative study of patient experience of virtual urgent care in Ontario

**DOI:** 10.1371/journal.pone.0285468

**Published:** 2023-09-22

**Authors:** Katie N. Dainty, M. Bianca Seaton, Justin N. Hall, Shawn Mondoux, Lency Abraham, Joy McCarron, Jean-Eric Tarride, Shelley L. McLeod

**Affiliations:** 1 North York General Hospital, Toronto, Canada; 2 Institute of Health Policy, Management and Evaluation, University of Toronto, Toronto, Canada; 3 Department of Emergency Medicine, Sunnybrook Health Sciences Centre, Toronto, Canada; 4 Division of Emergency Medicine, Temerty Faculty of Medicine, University of Toronto, Toronto, Canada; 5 Department of Emergency Medicine, St. Joseph’s Healthcare Hamilton, Hamilton, Canada; 6 Faculty of Health Sciences, McMaster University, Hamilton, Canada; 7 Ontario Health, Toronto, Canada; 8 Department of Health Research Methods, Evidence, And Impact, Faculty of Health Sciences, McMaster University, Hamilton, Canada; 9 Schwartz/Reisman Emergency Medicine Institute (SREMI), Sinai Health System, Toronto, Canada; 10 Department of Family and Community Medicine, Temerty Faculty of Medicine, University of Toronto, Toronto, Canada; Aalborg University and Aalborg University Hospital, DENMARK

## Abstract

**Introduction:**

In response to the COVID-19 pandemic, the Ontario Ministry of Health introduced a pilot program of 14 virtual urgent care (VUC) initiatives across the province to encourage physical distancing and provision of care by telephone and video-enabled visits. The implementation of the VUC pilot is currently being evaluated by an external academic team. The objective of this study was to understand patient experiences with VUC to determine barriers and facilitators to optimal virtual care as it rapidly expands during the current pandemic and beyond.

**Method:**

The qualitative component of the evaluation used one-on-one telephone interviews with patients, families, providers, and program administrators as the main method of data collection. Patient and family participants were invited to participate by the triage nurse after their VUC visit. Data analysis, using thematic analysis, occurred in conjunction with data collection to monitor emerging themes and areas for further exploration.

**Results:**

Between April and October 2021, we completed 14 patient and/or family interviews from a representative cross-section of 6 pilot sites. Participants had a range of presenting complaints including infection, injury, medication side effects, and abdominal pain. The vast majority of participants were female (90%), and 70% were VUC patients themselves. Our analysis identified three key themes in the data which characterise patient and family member experience with VUC: a) emphasis on access to the ED; b) efficiency and quality of care; c) obtaining reassurance and next steps.

**Conclusion:**

Virtual care options are valued by patients and families; however, the nature of care needed by those accessing VUC and who can best provide that care needs to be evaluated to position it for sustainability. Understanding how virtual care performs from both a provider and patient perspective during the current crisis has implications for designing alternative care options beyond the COVID-19 pandemic.

## Background

At the beginning of the COVID-19 pandemic in March 2020, emergency department (ED) volumes across Canada decreased by up to 50% due to fears of contracting the disease [1–3]. The Canadian Institute for Healthcare Information (CIHI) reported a 49% decrease in ED volumes after travel restrictions were introduced in March of 2020 [2]. The biggest drop in ED visits occurred during Wave 1, with almost 25,000 fewer visits—or about half the usual number—in mid-April 2020 [2]. The drop has been largely attributed to the way people interpreted public health restrictions and their fear of contracting COVID-19 at the hospital.

In response to the pandemic, the Ontario Ministry of Health released guidelines advising against in-person patient care in non-urgent situations and directed clinicians to transition the delivery of care to telephone or video-enabled virtual visits [4]. Virtual care has been defined as *“any interaction between patients and/or members of their circle of care*, *occurring remotely*, *using any forms of communication or information technologies*, *with the aim of facilitating or maximizing the quality and effectiveness of patient care”* [5]. Many professional Colleges encouraged their members to use virtual care whenever possible to minimize the risk of exposure amongst their patients, especially those at higher risk of harm from COVID-19 infection [6, 7]. Accordingly, several hospitals and clinics in Ontario began offering virtual care options for ambulatory patient care, including some emergency medicine. In addition to aligning with social distancing guidelines, virtual emergency medicine ensures that patients continue to have access to medical guidance for urgent (but non-emergent) or potentially serious medical conditions.

As part of the COVID-19 pandemic response, the Ontario Ministry of Health approved up to $4 million to fund regionally coordinated, virtual urgent care (VUC) initiatives across the province in the fall of 2020. Ontario is Canada’s most populous province and has the highest healthcare provision budget [8]. This funding was intended to support urgent care of patients with low acuity issues and reduce the need for face-to-face contact whenever possible. The project goals were (a) to enable patients to access urgent care virtually for lower acuity health issues or concerns within a specified time period, thereby avoiding unnecessary in-person ED visits; and (b) to support pandemic-related restrictions by providing patients with a virtual option to access ED care if they were concerned about exposure from presenting in person, thereby avoiding treatment delays for urgent conditions. Fourteen initiatives, mostly led by hospital emergency departments were eventually funded after going through the application and selection process.

Given that patient experience is increasingly being seen as an independent dimension of health-care quality along with clinical effectiveness and patient safety [9, 10], we need to understand factors associated with positive and negative patient experiences specific to the provision of optimal virtual urgent care during the COVID-19 pandemic and beyond. Accordingly, the objective of this study was to explore challenges and opportunities in the provision of VUC during the pandemic from the patient and family perspective, and to determine if there were any modifiable factors that could be leveraged to optimize virtual care in the future.

## Methods

### Study design

This was a qualitative descriptive study [11] using one-on-one interviews as the method of data collection. Institutional review board approval was obtained by the human research ethics board of XXXX Hospital (REB #21–0007). All participants were given time to review the project information letter and provided verbal consent prior to the start of data collection. This manuscript was prepared in accordance with the COREQ guidelines for qualitative research reporting [12].

### Participant recruitment

Patients or family members who could speak or read English (or understand with help) who had attended a VUC appointment with a participating pilot site between March 1 and September 30, 2021, were eligible to participate in this study. The interviews were conducted in English due to time and resource constraints. Patients and families were asked for consent to be contacted for a research interview about their experience with the virtual visit at the time of triage in a sample of selected sites participating in the larger provincial pilot project. The cohort of 6 sites selected for recruitment represented different geographic locations (urban, rural/large catchment area, northern) and models of care (nurse-led triage vs. self-screening).

The sample size for this study was determined by thematic saturation. This was defined as the point at which investigators agreed that a variety of viewpoints were accounted for, and successive interviews would provide no new insight into relevant themes [13, 14].

### Data collection

Patients and family members who consented to be contacted for an interview were contacted by a research coordinator and formally invited to participate. Those who agreed were sent a copy of the project information letter and consent form to review. Interviews were conducted by telephone or the Zoom^™^ virtual meeting platform to support social distancing restrictions and avoid geographic bias in recruitment.

Interviews were conducted by one of two PhD trained qualitative researchers (KND or MBS) and followed a semi-structured format using an interview guide informed by the study aim and addressing key topics (S1 Appendix) In the case of patients and families, this included understanding of the patient’s functional status, health concerns, and experience of the VUC visit. The selection of follow-up questions, question order, and phrasing varied according to each participant’s narrative, which enabled the emergence of participant-led accounts, reflecting their varied histories, modes of expression, and foci of experience. All interviews were audio-taped and transcribed verbatim by an external transcription service and data was managed using NVivo qualitative software [NVivo 12; QSR International Pty Ltd]. The interview transcripts were supplemented with field notes to collect data that could not be captured on audiotape (e.g., dynamics, emotional aspects, contextual factors).

### Data analysis

In keeping with the iterative process of qualitative methodology, data analysis occurred in conjunction with data collection to continuously monitor emerging themes and general areas for further exploration [15]. Data analysis was led by two team members with extensive qualitative experience (KND and MBS) and reviewed with the research team at various timepoints. Data was analyzed using a standard thematic analysis approach [16] which enables the identification of patterns of experience and meaning across the whole sample. Interview sections that reflect key areas of interest were extracted and collated during the initial coding process.

We then used the emergent codes to guide a de-novo analysis of the entire corpus for overarching sub-themes and used NVivo to record which subthemes occurred in each interview, ensuring their accurate representation in the analysis. Subthemes that express similar experiential patterns were brought together to build core descriptive which were represented across the data.

We employed the following techniques to support the analytic rigor and trustworthiness of our analysis: comparison of coding between analysts, seeking alternative explanations for the data during development of the final analytic framework, and interrogating the coherence of interpretations through discussion with the research team [17].

## Results

Between April and October 2021, we completed 14 patient and/or family interviews from a representative cross-section of 6 pilot sites. Participants had a range of presenting complaints including infection, injury, medication side effects, and abdominal pain. The vast majority of participants were female (90%), and 70% were VUC patients themselves. The remaining 30% were parents of children or family members of patients who attended the VUC consultation with the patient. Our analysis identified three key themes in the data which characterise patient and family member experience with VUC: a) emphasis on access to the ED; b) efficiency and quality of care; c) obtaining reassurance and next steps.

### Emphasis on access to the ED

Many of the study participants we spoke to immediately noted how much better they felt not having to physically go to the ED during the pandemic. Patients felt they needed to be seen by a health care provider, but often could not get timely access to primary care because of pandemic related office closures. At the same time, they were very concerned about the possibility of contracting COVID-19 while waiting in a crowded ED waiting room, potentially for something that was urgent but not emergent. They explained to us that VUC was able to provide an alternative option to delaying care.

While the pandemic certainly created a heightened fear of COVID-19 infection exposure, participants also talked about the convenience of VUC outside the pandemic, particularly for those with mobility issues or small children. We repeatedly heard that patients struggled with whether or not to go to the ED for what may be a minor, yet time-sensitive, health concern.

“Like given that there is the pandemic, right? It’s nice not to have that possibility of exposure. The pandemic aside, you know, going to the emergency room, there are sick people. So, you can get anything. And then on top of that, you know, people who have kids are more limited with transportation. It’s just nice not to have to deal with the other issues just to get yourself there”.

[P9 FM]

### Efficiency and quality of care

Although VUC was operationalized quite quickly during the start of the pandemic, many participants discussed being very impressed with the quality of the virtual care interaction. They cited not only convenience, but felt they actually received more efficient care and more focused attention from the physician than what they typically received during in-person visits to the ED. They told us they felt “well cared for” and described being led through a virtual examination, thorough question and answer consultation, and having a detailed discussion about their health issues and concerns.

“The doctor was very nice. Usually, when I go in person, I sometimes feel that the doctor is very rushed, and they have to go room to room at like a very fast speed. But I felt like the doctor maybe in the sense that it was virtual, and they didn’t have to travel much, they were able to spend a lot of quality time going through asking me questions that were very relevant…. they just took a lot of time to make sure that I wasn’t really worried about something that I had to be aware about or something like that. So, it was nice. It was a good experience.”

[P6 PT]

Participants were also very pleased that they were able to get specific treatment advice or a medication prescription from the appointment, not just general advice, or an automatic recommendation to go to the ED. Several compared it to previous telehealth experiences, which were not as helpful.

“…when you talk to these, like the on-call nurses, I always am skeptical because I always get the same response–“well, you should probably call the emergency and go. Or you should call an ambulance and go”. So, it’s not like they’re giving you medical advice. They are just sort of covering themselves. But with this I was surprised it was actually giving me a spot to speak to an actual ED physician. So overall the experience was very positive. I explained to her the situation. She said yes, it is a little bit too weak of a type of antibiotic, so she emailed me a prescription, which my wife sent to the pharmacy, and I started that same night on these new antibiotic pills.”

[P4 PT]

“One of the things that I was also impressed about is that I got an email after–a PDF email was actually sent to me with the doctor’s notes. And like a record, with the doctor’s name on it. It was a really nice PDF with the hospital logo. It was very clean and easy to understand. And that was emailed to be right after the appointment, so I love that.”

[P9 PT]

### Obtaining reassurance and next steps

All the study participants we interviewed were reassured they could access a health care provider and discuss their health concerns or symptoms to either receive treatment or determine if a more in depth, in-person ED visit was necessary. In many cases they were able to resolve their issue with the provider during the virtual appointment with medical advice and/or a prescription. Participants who required further care received a treatment plan and/or referral to their local ED and highlighted how much they valued understanding the next steps to be taken to address their care needs.

“You know she [the nurse] was just kind and caring letting me know, “okay, that’s pretty bad. We’re going to have the doctor look at this”. And kind of telling me what the outcomes would most likely be so that I didn’t sit there and panic. Because by the time I had eyes on it, and it was bright green, and, you know, pussing a little bit. I was like okay, am I going to lose my arm?? I was a little freaked out. But they were very reassuring, you know, that I had taken the right approach to call.”

[P5 PT]

For those that were ultimately referred to the physical ED, it was also reassuring to know they would be triaged when they got there, and the ED staff would be expecting them. This made the idea of having to go in more palatable; the “warm handover” not only made for more efficient care but also made the patients feel reassured they had not wasted time or resources in the ED.

## Discussion

Our findings suggest that patients who accessed VUC from sites participating in the provincial VUC pilot program were highly satisfied with the care they received. It was especially beneficial during the COVID-19 pandemic as it allowed patients to avoid exposing themselves to unnecessary infection risk and waiting in overcrowded ED waiting rooms for low acuity concerns. Patients shared that they were very impressed with the efficiency of VUC and were reassured by talking to a skilled health care provider about the most appropriate next steps in their care/treatment.

These findings are important for two reasons: 1) they articulate the value patients put on easily accessible, efficient, high-quality alternatives for urgent care, both within and outside the pandemic situation and 2) they are well aligned with the domains our team developed regarding patient perspectives of important outcomes following care and discharge from the ED [18]. Although patients present to the ED for different reasons, our PROM-ED research highlighted a common set of outcomes valued by patients that can be grouped under four core domains: 1) understanding the causes and implications of symptoms; 2) reassurance in regard to the worry and distress that brought the patients to the ED; 3) achieving symptom relief; and 4) having a plan to manage their symptoms, resolve their issue or pursue the diagnostic process.

With or without the COVID-19 pandemic, hospital-based ED care is at a breaking point [19]. This combined with challenges with access to timely primary care [20, 21], increases the potential for delayed or missed presentations of urgent care situations. The data presented here supports the desire for alternative models of urgent care which are more focused on meeting the needs of the patients in an efficient and reassuring manner, rather than continuing to maintain a traditional, provider-centred way of organizing care. Virtual urgent care appears to be a very acceptable option for those who feel they need urgent, but not *emergent*, care or who have a reasonable need to be evaluated to determine the need for more advanced in-person diagnostics or care.

## Limitations

This qualitative study looked at the patient and family experience of a pilot VUC program in a single Canadian province. We recruited participants from several different hospital sites (e.g., urban, rural, adult, pediatric) as well as a variety of clinical reasons for using VUC so we feel that our sample is representative of those patients and families who accessed the program.

We were only able to conduct the interviews in English due to resource constraints. While not ideal, 92.4% of patients presenting to the sites involved in the provincial VUC pilot program primarily spoke English. It is possible that non-English speaking patients or family members may have had a different experience than what is reflected in our analysis.

Provider perspectives on delivering virtual urgent care are an important part of understanding the impact of such a service. We also interviewed physicians, nurses and administrators who participated in the program and the findings will be reported in a separate manuscript.

## Conclusions

Virtual urgent care is seen as a valued care option for patients and their families, both during a pandemic and beyond. Patients believed they received efficient and attentive care and felt reassured in being evaluated before making a potentially unnecessary trip to what they perceive to be an overtaxed ED. From the patient and family perspective, VUC is a viable care option that should be part of future health care planning.

## Supporting information

S1 Appendix Patient interview guide. (DOCX)
