# Supplementary material for: “It saved me from the emergency department”: A qualitative study of patient experience of virtual urgent care in Ontario
Source: PLoS One. 2023 Sep 22;18(9):e0285468. doi: 10.1371/journal.pone.0285468 (PMC10516408; doi:10.1371/journal.pone.0285468)
Supplement: S1 Appendix — (DOCX) [file pone.0285468.s001.docx]

**APPENDIX A - Patient Interview Guide**

Introduction: The purpose of this study is to learn about your experience with your virtual emergency department visit at {insert site}. I’m particularly interested in hearing about what you thought about how it went and if and how it could have been improved.

- To start, can you tell me a little bit about yourself and your virtual ED visit?
  1. Did you speak to the doctor on the phone or by video?
  2. What made you think to try the virtual ED clinic?
- What were your initial thoughts when you heard you would be talking to the doctor virtually?
- Can you tell me about how you thought the appointment went?
- Was there anything that you thought could have been better about the appointment?
- How did you feel when you left the virtual ED appointment?
  1. PROMS prompts - happy with the outcome, reassured, have a plan?
- Would you recommend a virtual ED visit to your friends and family?
  1. Why? Why not? As appropriate
- Is there anything else you would like to tell me today about your experience with virtual urgent care?
